# Bridging the Global Technology Gap in Neurosurgery: Disparities in Access to Advanced Tools for Brain Tumor Resection

**DOI:** 10.1227/neuprac.0000000000000090

**Published:** 2024-04-25

**Authors:** Jose E. Valerio, Felipe Ramirez-Velandia, Maria Paula Fernandez-Gomez, Noe S. Rea, Andres M. Alvarez-Pinzon

**Affiliations:** * Department of Neurological Surgery, Palmetto General Hospital, Miami, Florida, USA;; ‡ Neurosurgery Oncology Center of Excellence, Department of Neurosurgery, Miami Neuroscience Center at Larkin, South Miami, Florida, USA;; § GW School of Business, The George Washington University, Washington, District of Columbia, USA;; ‖ Clinical Research Associate, Latino America Valerio Foundation, Weston, Florida, USA;; ¶ The Institute of Neuroscience of Castilla y León (INCYL), Cancer Neuroscience, University of Salamanca (USAL), Salamanca, Spain; # Stanford LEAD Program, Graduate School of Business, Stanford University, Palo Alto, California, USA;; ** Institute for Human Health and Disease Intervention (I-HEALTH), Florida Atlantic University, Jupiter, Florida, USA

**Keywords:** Global neurosurgery, Technology, Innovation, Research, Neuronavigation, Virtual reality, Augmented reality, Robotics, High-grade gliomas, Cost

## Abstract

**BACKGROUND AND OBJECTIVES:**

The advent of advanced technologies has brought unprecedented precision and efficacy to neurosurgical procedures for brain tumor resection. Despite the remarkable progress, disparities in technology access across different nations persist, creating significant challenges in providing equitable neurosurgical care. The purpose of the following work was to comprehensively analyze the existing disparities in access to innovative neurosurgical technologies and the impact of such disparities on patient outcomes and research. We seek to shed light on the extent of the problem, the underlying causes, and propose strategies for mitigating these disparities.

**METHODS:**

A systematic review of published articles, including clinical studies, reports, and healthcare infrastructure assessments, was conducted to gather data on the availability and utilization of advanced neurosurgical technologies in various countries.

**RESULTS:**

Disparities in technology access in neurosurgery are evident, with high-income countries benefiting from widespread implementation, while low- and middle-income countries face significant challenges in technology adoption. These disparities contribute to variations in surgical outcomes and patient experiences. The root causes of these disparities encompass financial constraints, inadequate infrastructure, and insufficient training and expertise.

**CONCLUSION:**

Disparities in access to advanced neurosurgical technology remain a critical concern in global neurosurgery. Bridging this gap is essential to ensure that all patients, regardless of their geographic location, can benefit from the advancements in neurosurgical care. A concerted effort involving governments, healthcare institutions, and the international community is required to achieve this goal, advancing the quality of care for patients with brain tumors worldwide.

**ABBREVIATIONS:**
AC: awake craniotomy
AI: artificial intelligence
AR: augmented reality
HICs: high-income countries
iMRI: intraoperative magnetic resonance imaging
iUS: intraoperative ultrasound
LMICs: low-middle income countries
VR: virtual reality.

Brain tumors impose a significant burden on the healthcare systems, due to their high mortality rate, disability, and adverse impact on quality of life.^1^ In response, the field of neurological surgery has been steadfast in developing advanced technologies pursuing maximally safe resection of brain lesions.^2^

The advent of these technologies, including preoperative augmented reality (AR) and virtual reality (VR) planning, as well as intraoperative technologies such as neuronavigation systems, ultrasound, and intraoperative MRI, has brought unprecedented precision and improve overall survival of patients with high-grade gliomas.^3-7^ Despite the remarkable progress, disparities in technology access across different nations persist, creating significant challenges in providing equitable neurosurgical care.^8^ It has been estimated than 90% of scanners worldwide are concentrated in high-income countries (HICs); therefore, around 70% of the world's population have almost no MRI access.^9^ Moreover, neuronavigation requires expensive specialized hardware and specific imaging protocols, as well as its high retail price, limiting its availability worldwide.^10^ Although the cost of these technologies is high, much higher is the costs associated with the care of patients with high-grade gliomas, reported to be as high as $138 767/patient in a span of 6 months.^11^

This review article aims to analyze the existing disparities in access to innovative neurosurgical technologies and the impact of such disparities on patient outcomes. We seek to shed light on the extent of the problem, the underlying causes, and propose strategies for mitigating these disparities.

## METHODS

A systematic review was conducted in multiple databases, including MEDLINE (PubMed and Ovid), Embase, Scopus, and Web of Science including studies published from January 1, 2021, to October 25, 2023, using the following terms: “augmented reality,” “virtual reality,” “neuronavigation,” “robotics,” “brain mapping,” “iMRI,” “iUS,” “artificial intelligence,” “research,” “neurosurgery,” “countries,” “resource poor,” “developing country,” and “income.” Boolean logic (“AND” and “OR”) was implemented in the search concepts, with the use of advanced Medline Subject Headings. The included databases enhance the diversity of sources and minimize the likelihood of missing information in the gray literature. Inclusion criteria were clinical studies and reports evaluating technological innovations used to maximize tumor resection and improve outcomes in the field of neuro-oncology. We excluded commentaries, letters to the editor, case reports, and articles written in languages other than English or Spanish. Two independently reviewers (F.R.-V., N.R.) screened the titles and abstracts, and disagreements were resolved by the senior author in this paper (A.A.P.). Full text was then reviewed for data extraction and organization on a predecided Excel spreadsheet. A comprehensive flowchart of selection process of the articles included in this review is shown in Figure. Our review was not registered in PROSPERO.

**FIGURE.**
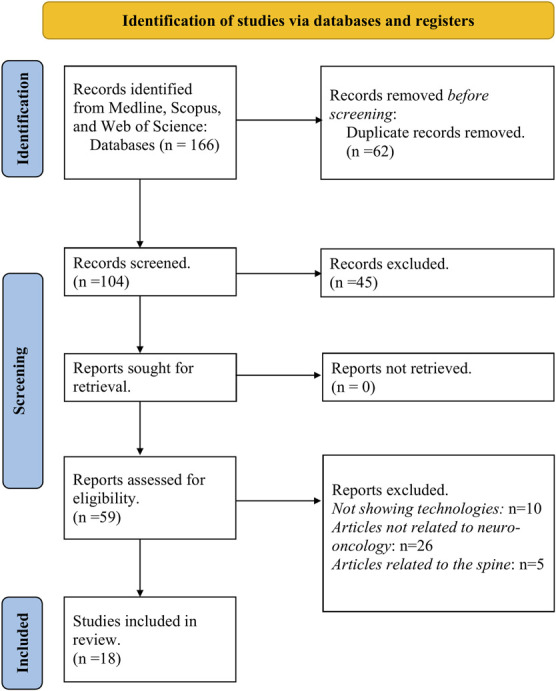
The Preferred Reporting Items for Systematic Reviews and Meta-Analyses flowchart of the selection process of the articles included in this literature review. The initial search strategy retrieved 166 manuscripts from MEDLINE, Scopus, and Web of Science. Subsequently, 62 duplicate records were identified and removed. Furthermore, 45 manuscripts were excluded as they did not meet the predefined inclusion criteria, specifically focusing on studies evaluating the impact or cost of technological innovations in the field of neuro-oncology. From the remaining 59 manuscripts selected for full-text review, we excluded 10 papers that lacked a focus on technologies and primarily concentrated on clinical outcomes without delineating the associated benefits and strategies used to minimize technology gaps. In addition, 26 papers were found to be related to technological strategies but were not directly tied to neuro-oncology, and 5 were specific to spine-related topics. In the end, 18 articles met the criteria and were included in this review. The summarized findings are presented in Table 1.

## RESULTS

We found a total of 18 articles evaluating technological disparities in the literature, with evident difference among the evaluated countries. The summary of the included studies is presented in Table 1, and the strengths and pitfalls of each technology are presented in Table 2.

**TABLE 1.** Summary of the Articles Included in This Review, Strength, and Points of Intervention

Reference	Innovation	Key findings	Potential area of intervention/strengths
Vasan et al, 2020^14^	Medical devices	LMICs lack of trained personnel, limitations with infrastructure, and the lack of spare parts or support for equipment.	International collaboration
Perez-Chadid et al, 2023^15^	Imaging guidance systems	Young neurosurgeons from Latin American and Caribbean Countries have access to imaging modalities in, but guided systems are only available in 27.5% of institutions.	International collaboration
Kanmounye et al, 2021^16^	Imaging guidance systems	Young neurosurgeons and residents from Africa report barriers to education, research, and practice, with only 24.1% of institutions having image-guided systems.	Sustainable training programs; technology sharing and mentorship
Gnanakumar et al, 2020^17^	Equipment and service access	MRI is available in 89.8 % of LMICs and 66.7% of low-income countries. 33.7% of LMICs and 12.8% of low-income countries have image-guided systems.	Technology sharing and mentorship
Rodseth et al, 2017^18^	Navigation system	Low cost ($3600 USD) navigation system with high accuracy (positional error of 1.2 mm and orientational error of 0.3°) as well as consistency.	Increases affordability in LMICs
Léger et al, 2022^19^	Neuronavigation system	Low cost ($10 000 USD) navigation system can be easily used by neurosurgeons and nursing staff.	Increases affordability in LMICs
Kaale et al, 2021^20^	iUS	The use of iUS in Tazania was useful identifying the residual tumor during craniotomies or laminectomies. Postoperative imaging correlated with the findings observed through postdural closure ultrasound.	Access to intraoperative technologies in LMICs
Cho, 2023^22^	Portable MRI	A $250 000 low-field scanner has been developed to provide a portable imaging system. This scanner is currently under evaluation in Uganda.	Access to intraoperative technologies in LMICs
Gosal et al, 2021^24^	Simulation/virtual reality	A 3D volume rendering system has been used in India for deep-seated lesions, helping identify the ideal surgical corridor and residual tumor.	Access to intraoperative technologies in LMICs
de Almeida et al, 2022^25^	Neuronavigation/virtual reality	The NeuroKeypoint app has been developed in Brazil for neuronavigation, demonstrating a mean target error of 2.6 ± 1.6 mm for identifying anatomic landmarks in replicas of human scalp.	Access to intraoperative technologies in LMICs
Kuang et al, 2023^26^	Artificial intelligence	ChatGPT exhibits limitations in neurosurgery as its responses are highly dependent on the user's input, relying on patterns and repetition. The results suggest inaccurate responses and outdated information that may lack proper referencing.	Guide for primary care providers of general practice in neurosurgery
Sutherland et al, 2015^29^	Robotics	NeuroArm, an image-guided robot developed in Canada for surgical resection of gliomas, was used in 18 patients demonstrating advantages navigating narrow surgical corridors, improving surgical ergonomics, precision, and accuracy.	Sustainable training programs; technology sharing and mentorship
Brower, 2002^31^	Robotics	Telemedicine has now expanded to the field of telerobotics, bringing a positive effect to globalization. In September 2001, a groundbreaking transatlantic telesurgery in Strasbourg, France, demonstrated the feasibility of remote surgery, now possible with advancements in technology and robotics.	Technology sharing and mentorship
Mofatteh et al, 2023^32^	Awake craniotomy	The challenges in performing awake craniotomy in Africa include infrastructure limitations, resource constraints, limited access to imaging, and human resource limitations, collectively affecting patient care and outcomes.	International collaboration
Figueredo et al, 2023^33^	Awake craniotomy	This comprehensive review of awake craniotomy in Latin America reported that 34.3% of cases achieved gross-total resection, 62.9% using an Asleep-Awake-Asleep protocol and 14.8% using Awake-Awake-Awake; the mean postsurgery discharge time was 68 h. Challenges, including limited training and infrastructure, were addressed through strategies such as specialized center training, sponsorships, and multidisciplinary collaborations.	International collaboration; resource allocation
Albuquerque et al, 2021^34^	Awake craniotomy	Challenges in implementing awake craniotomy program in LMICs include a lack of access to neuronavigation, limited resources, equipment availability and surgical scheduling, difficulties offering a neurocognitive evaluation.	International collaboration; resource allocation
Khan et al, 2017^35^	Awake craniotomy	Awake craniotomy offers significant benefits in LMICs as it can decrease the healthcare-associated costs of patients with high-grade gliomas and the need for rehab. However, challenges persist due to limited resources, the requirement of neuroanesthesia training, and appropriate patient selection	International collaboration; resource allocation
Tansley et al, 2016^38^	Simulation	A simulation training system applied to 26 postgraduate surgical trainees from Rwanda revealed improvement in the objective structured assessment of technical skill by 6.1 points, with a higher increase for junior practitioners.	Sustainable training programs; international collaboration

iUS, intraoperative ultrasound; LMICs, low- and middle-income countries.

**TABLE 2.** Summary of the Strengths and Pitfalls of LMICs and HICs in the Different Technological Innovations Identified in the Selected Articles

Technologies	LMICs	HICs
Neuronavigation		
Strengths	• Development of low-cost neuro-navigation systems.	• Access to advanced neuronavigation technologies with high accuracy.
	• Use of intraoperative ultrasound as a cost-effective alternative	• Accessibility to intraoperative MRI for enhanced outcomes.
Pitfalls	• Limited access due to cost and lack of infrastructure.	• High cost of neuronavigation equipment.
	• Challenges in training personnel for effective use.	• Limited access in remote areas due to high equipment costs.
	• Inequality in global access to advanced technologies.	• Time consuming, requires more personnel
AR/VR		
Strengths	• Adoption of affordable 3D reconstruction technologies from preoperative computed tomography/MRI.	• Adoption of AR/VR technologies for surgical planning.
	• Development of low-cost AR apps for neuronavigation.	• Integration of advanced AR/VR systems for neuronavigation and
Pitfalls	• Limited access	• Dependency on underlying image-guided systems. • Technical limitations. • Requires familiarity with the software
AI		
Strengths	• AI platforms for image analysis aid in remote areas with shortages.	• Advanced AI platforms for community hospitals
	• Potential use as a source for general understanding in neurosurgery.	
Pitfalls	• Limited comprehension of technical terminology in neurosurgery.	• Poor performance in providing references/resources.
		• Challenges in using AI for detailed treatment risk analysis.
Robotics		
Strengths	• Telerobotics enabling remote surgeries. • Potential for overcoming geographical barriers.	• Robotic neurosurgery enhancing precision and safety • Integration of robotic systems like NeuroArm for enhanced surgery.
Pitfalls	• Limited access • Very expensive technology • Complex logistics and challenges in remote procedures.	• Higher costs and learning curve associated with robotic systems. • Concerns about internet reliability and security in telerobotics.
Awake craniotomy		
Strengths	• Feasibility in low-resource settings.	• Widespread use in advanced neurosurgical facilities for treating tumor in eloquent areas.
	• Potential to minimize hospital costs and human resources.	• Less postoperative neurological deficit.
Pitfalls	• Limited infrastructure and funding	• Requirement for advanced facilities and trained personnel.
Simulation		
Strengths	• Controlled learning environments for training neurosurgeons in LMIC.	• Widely used in high-income countries for hands-on practice.
		• Realism and confidence-building in surgical skills.
Pitfalls	• Scarcity of simulators and instructors in LMIC. • Need for international collaboration to address training gaps.	• Not readily available at most centers

3D, 3-dimensional; AI, artificial intelligence; AR/VR, augmented and virtual reality; HICs, high income countries; LMICs, low-and-middle income countries.

### Intraoperative Technologies

The origins of neuronavigation can be traced back to 1949 with the development of the Leksell frame.^12^ The frameless navigation system was introduced in 1986 and has become a widely used technology for neuronavigation in tumor resection procedures, incorporating additional technologies over years to enhance overall accuracy.^13^ The documented benefits of neuronavigation, including improved tumor resection and overall survival, are well-established in the literature.^3-5^

However, a global gap exists in accessing neuronavigation systems, particularly in community hospitals of low-middle income countries (LMICs). The World Health Organization estimates that 70% of developed countries equipment does not function in LMICs hospitals due to a lack of trained personnel and limited infrastructure.^14^ Moreover, surveys among young neurosurgeons in Latin America and Africa have revealed that only 24.1% to 25.7% of providers have access to imaging guidance systems.^15,16^ Not surprisingly, the World Federation of Neurosurgery Societies reveals that only 33.7% of institutions in LMICs have an available navigation system in the operating room for their use.^17^

There is a high cost for neuronavigation equipments, which limits the affordability for LMICs. It has been estimated that the cost of a system is $60 000 but may vary depending on the vendor and country.^10^ To address this cost concerns, institutions around the world have developed cheaper systems. Rodseth et al^18^ proposed a $3600 USD system with an Opti track trio tripod, a 3-dimensional (3D) Plug in, and a costume software reporting a positional error of 1.2 mm and orientational error of 0.3°. More recently, a prototype of an easy intraoperative system called the NousNav has been developed; this system can be used by either the practicing clinician or the nurse, and it costs <$10 000 USD.^19^

Alternatively, a low-cost and efficacious alternative has been the adoption of intraoperative ultrasound (iUS) for tumor resection, readily available in most institutions' worldwide. In fact, some centers in Tanzania have used iUS for both spinal and cranial tumor resection procedures, reporting additional benefits in identifying residual tumor and improving the extent of tumor resection.^20^ However, training in these imaging techniques is essential, as challenges related to spatial resolution, orientation, image quality, and lack of experience have led to ultrasound frequently being replaced by computed tomography–based and magnetic resonance–based navigation systems.

More recently, institutions have been using intraoperative magnetic resonance imaging (iMRI) to improve the extent of resection of high-grade gliomas. Recent analyses comparing neuronavigation with iMRI have shown that the use of iMRI maximizes resection and provides an average of 1.5 additional months of progression-free survival.^6^ This technology has proven to be cost-effective when analyzing the costs related to the high morbidity of high-grade gliomas.^21^ However, reported costs of iMRI can reach up to 1.5 million USD, making it inaccessible to 70% of the world's population.

Alternatively, substantial efforts have been made in the development of portable magnetic MRI scanners. The Swoop scanner (Hyperfine®), which costs about $250 000, provides brain imaging without the need for conventional, large, and expensive superconducting electromagnets.^22^ This low-field scanner, approved by the US Food and Drug Administration, has potential applications in diverse settings. Despite the lower resolution of this scanner, The CURE Children's Hospital of Uganda is developing a project to test the utility of low-field MRI in the developing world. The hospital plans to compare the low-field MRI scanner developed by Johnes Obungoloch, a biomedical engineer at Mbarara University of Science and Technology, with the Swoop technology. Other similar devices are on the horizon, challenging traditional high-field MRI practices and providing solutions to the lack of access to neuroimaging in remote and LMICs.

### AR/VR

The integration of VR and AR technologies in Neurosurgery Oncology is a promising tool for preoperative planning aimed at improving tumor resection.^23^ However, the widespread adoption of VR and AR systems in LMICs faces challenges, related to the requirement of compatible image-guided systems, which may not be available in resource-constrained settings. Consequently, alternative approaches have been explored to leverage existing resources, such as using 3D reconstruction techniques based on computed tomography and MRI images. At a single center in India, the utilization of the 3D volume-rendering technique (RadiAnt) reported benefits in the resection of deep-seated gliomas by providing a precise assessment of the residual tumor location and extent of resection.^24^

Moreover, an institution in Brazil has developed an easy-to-use low-cost AR app for neuronavigation, reporting a mean target error of 2.6 ± 1.6 mm.^25^ The app (NeuroKeypoint, App Store) has an integrated system using the Swift programming language. It includes an interface for entering image coordinates and a Horos plugin for QR code input, obtaining information from predefined AR markers attached to electrodes on the patient's head. This approach enables precise visualization and assessment of tumor morphology and its relationship with adjacent structures, allowing to strategize optimal resection plans.

### Artificial Intelligence

Artificial Intelligence (AI) platforms have emerged as transformative tools aimed at augmenting the capabilities of physicians in image interpretation, diagnosis of surgical conditions, and informing treatment decisions. These technological advancements hold significant promise, particularly in healthcare institutions grappling with personnel shortages, a common challenge observed in remote rural areas or LMICs. Among the plethora of AI platforms currently under development, ChatGPT stands out as one of the most widely recognized. Studies assessing its efficacy in the context of neurosurgical applications have yielded encouraging results, indicating its potential to enhance clinical decision-making processes.^26^ However, AI platforms like ChatGPT have limitations in understanding specialized medical terminology and providing accurate responses tailored to neurosurgical practice.^27^ Thus, AI platforms, while offering invaluable support to healthcare providers, require continuous refinement to seamlessly integrate into neurosurgery.

Beyond assisting medical practitioners, AI platforms are gaining recognition for their capacity to expand healthcare and clinical research accessibility.^26,27^ AI has enabled healthcare providers to conduct remote patient monitoring and provide timely interventions and expert guidance in clinical research, regardless of geographical constraints. Regions that do not have access to specialists can receive neurosurgical expertise, which can contribute to the decentralization of health care and the reduction of health disparities. Furthermore, AI-powered educational platforms, using machine learning algorithms, have proven to be invaluable resources for neurosurgical training and capacity building.^27^

### Robotics

Robotic neurosurgery has demonstrated efficacy for tumor resection in developed countries.^28^ A robot developed in Canada (NeuroArm) has integrated an image-guided system designed to enhance surgical ergonomics, patient safety, precision, and accuracy.^29^ This robot includes a specialized tremor filter that enables smooth and precise movements of robotic arms. Moreover, the use of telerobotics and 5G networks has enabled physicians to conduct surgeries in distant locations across various surgical specialties.^30^ Notably, in 2001, surgeons in New York successfully removed a patient's gallbladder in France using a remote-controlled robotic system, known as the Lindbergh operation.^31^ However, the adoption of this innovative practice demands a substantial investment and familiarity. Complex logistics associated with remote procedures, coupled with concerns about internet reliability, bandwidth, speed, and security, have hindered widespread implementation of telerobotic surgeries so far, preventing it from becoming a common practice.

### Awake Craniotomy

Awake craniotomy (AC) has become increasingly common in advanced neurosurgical facilities, aiming to enhance surgical resection while minimizing the risk of injury to eloquent areas. Interestingly, AC can be safely performed in low-resource settings. In a comprehensive analysis of AC in Africa, the challenges encountered included a shortage of personnel and basic equipment, poor awareness of AC among patients, limited access to health care, and high procurement costs.^32^ Similarly, a comprehensive review in Latin America has revealed that the adoption of AC is hindered by challenges such as limited infrastructure, a shortage of trained personnel, and inadequate funding.^33^

AC requires intraoperative brain mapping, advanced imaging, neuromonitoring, specific anesthesia, and trained neuropsychologists, which are not readily available in LMICs.^34^ In other cohorts, AC has shortened hospital stay, ultimately minimizing the hospital costs and the human resources required for providing a comprehensive care for patients.^35,36^ With these benefits, LMICs have been interested in seeking funding for equipment, supplies, and training for surgical technicians and anesthesiologists.

### Simulation

The evolution of simulation platforms has created similar scenarios to practice complex neurosurgical procedures.^37^ These platforms provide students, residents, and fellows with a controlled learning environment that effectively diminishes the gap between the observation and the hands-on practice. Simulation training is primarily used in HICs, while some cohorts have explored its application in LMICs.^38^ However, there is absence of compelling evidence regarding the effectiveness of these programs in LMICs, where there may be shortage of simulators and personnel. In addition, since the expertise needed to instruct surgical techniques in simulated environments has predominantly evolved in wealthier regions, the scarcity of local instructors in LMICs may impede the efficacy of simulation training in these contexts.

## DISCUSSION

There has been incredible progress in neurosurgical technologies designed to improve outcomes for patients with brain tumor. Nonetheless, numerous disparities exist between HICs and LMICs. A wide range of technological advancements, including inexpensive and user-friendly neuronavigation systems, the incorporation of iUS, and the creation of portable MRI scanners, are discussed in the subsequent analysis. We additionally evaluate the potential implementation of telesurgery, as well as the obstacles associated with this methodology within the domain of neurosurgery. Following this, we shall expound on the 5 strategies that have garnered the highest reports of efficacy in mitigating technological disparities between HICs and LMICs.

### International Collaboration

Collaboration on an international scale has enabled LMICs to access to technological innovations in neurosurgery.^39^ A great example is Ethiopia, which stablished the first residency training program for neurosurgery in 2006, including international rotations. Currently, after 14 years of collaboration, the neurosurgeon density has increased by 20-fold.^40^ de Almeida et al^41^ suggested structured training programs, neurosurgical education mission trips, online surgical curriculum, and long distance mentorship for international collaboration. While all are interconnected, the most valuable is likely the widespread use of the internet and social networks. Electronic distance learning (eLearning) has gained widespread acceptance and is extensively used in the field of medicine.^42^ eLearning has shown to have positive educational outcomes and to be much cheaper compared with the expenditure of surgical trips. Moreover, social media platforms serve as effective communication tools, connecting neurosurgeons, trainees, and medical students worldwide.^39^ This teamwork can drive the development of universally applicable solutions, allowing connectivity, and fostering the relations established among different institutions around the globe.

### Resource Allocation

Equally as important as international connectivity is the equitable allocation of resources. Neurosurgical technologies require substantial financial investments, and identifying the optimal recipients can be challenging (equipment needs and volume).^43^ Sources can originate from local governments, international organizations, or research grants. The National Institute of Health is the world's largest supporter of biomedical research, and the Fogarty International Center, one of its 27 institutes, is dedicated to training scientists and enhancing research in LMICs; alternatively, the US Agency for International Development focuses on foreign assistance to developing countries.^44^ According to a retrospective analysis performed from 2007 to 2013, the National Institute of Health has funded over 22 projects with international collaboration, totaling of $31.3 million USD.^44^ Some of these funds have been allocated to scholarship programs, facilitating short-term study stays abroad aimed at improving and acquiring skills in affordable technologies.

### Sustainable Training Programs

Initiatives to train and mentor neurosurgeons in underprivileged regions are an option to overcome the gap in access to latest technologies. In fact, the University of Toronto has established a model for structured training wherein neurosurgeons from a LMIC temporarily participate in an academic training program in a HIC. This self-funded 1-year fellowship program, stablished since 2011, has trained 27 neurosurgeons from different countries, including 6 trainees from LMICs.^41^ Although it is a short training program, participants have reported great benefits in the long-term relationships. These connections have enabled trainees to update on the latest technological innovations and potential collaborations for research projects. Current practices incorporate telemedicine into the clinical follow-up of neurosurgical patients. This integration enables physicians from low- and middle-income countries to collaborate and participate in meetings and case discussions. Furthermore, training programs can be expanded to include sonographic imaging of the brain, as difficulties in implementing navigation with 3D ultrasonography are related to a lack of experience in its use due to poor orientation or improper technique. Similarly, anesthesiologists can gain expertise in the setting, requirements, and monitoring of awake surgery, which is one of the limitations of performing awake surgery in LMICs.

### Technology Sharing and Mentorship

Emerging trends in global surgical training have included the adoption of tele-mentoring and tele-proctoring during surgeries.^45^ Surgical tele-mentoring, consisting of an experienced surgeon guiding a less-experienced trainee, has shown promising results in improving the techniques of surgeons in rural areas.^46^ Similarly, tele-proctoring, which uses internet connectivity to broadcast in real time the surgery, establishes an audio and visual connection between surgeons in different locations, enabling real-time guidance without physical presence.^47^ The integration of immersive technologies could further enhance this field, allowing mentoring surgeons to demonstrate procedural steps and advice while the procedure is being performed.^48^ More interestingly, low cost devices have incorporated these technologies. The iPad-based platform Virtual Interactive Presence and Augmented Reality (VIPAR) the AR/VR headsets through smartphones, and the headsets by Google Cardboard have allowed mentoring surgeons to virtually extend their guidance into the display of the mentee surgeon.^49,50^ These technologies offer sustainable solutions for surgical training in resource-limited locations. Mentorship also offers invaluable support and guidance to aspiring neurosurgeons, as well as to trained professionals. Research opportunities through mentorship have opened significant avenues for medical trainees and professionals from LMICs to additional training in HICs.

### Public-Private Partnerships

Finally, the involvement of governments, international organizations, and the private sector has shown to enhance technology accessibility. A Public-Private Partnership model is a type of collaboration between public and private sectors, where the private sector provides funding and expertise to deliver public services or infrastructure projects.^51^ Public-private partnerships can lead to innovative financing models, accelerated research in minority groups, and the development of affordable technologies. Such collaborations can overcome financial barriers and accelerate the diffusion of innovative neurosurgical tools.^52^

### Limitations

During our systematic review, we recognize limitations affecting our findings. We mitigated potential publication bias by using multiple databases, minimized language bias by including only English and Spanish studies, and acknowledged possible geographical biases from focusing on specific regions. Despite these limitations, we offer an overview of the existing literature, and our findings align with those reported by other authors.

## CONCLUSION

The persistent disparities in access to advanced neurosurgical technology present a critical challenge in global health care, particularly important in neurosurgery, innovation, and research. Bridging this gap is essential to ensure that all patients, regardless of their geographic location, can benefit from the advancements in neurosurgical care. To achieve this, a collective effort involving governments, healthcare institutions, and the international community is essential. Through collaborative initiatives, we can bridge the gap, offering hope to patients with brain tumors and complex neurological conditions worldwide. By pooling resources, knowledge, and expertise, we hold the potential to transform the landscape of neurosurgical care, promising improved patient outcomes and fostering a global network of support, compassion, and excellence in the practice of neurosurgery.
